# Impact of Different Extraction Solvents on Phenolic Content and Antioxidant Potential of* Pinus densiflora* Bark Extract

**DOI:** 10.1155/2019/3520675

**Published:** 2019-07-29

**Authors:** Thamizhiniyan Venkatesan, Young-Woong Choi, Young-Kyoon Kim

**Affiliations:** Department of Forest Products and Biotechnology, College of Science and Technology, Kookmin University, 861-1 Chongnung-dong, Songbuk-gu, Seoul, 136-702, Republic of Korea

## Abstract

It is well established that various extraction factors, including the method, temperature, time, and solvent system, significantly influence the antioxidant quality of plant-derived products. Previously, we observed that extraction of* Pinus densiflora* bark (PDB) by the most common traditional Soxhlet method using water at two different temperature conditions 60°C and 100°C for 6-15 h noticeably altered their antioxidant quality. In this study, we examined the impact of different extraction solvents such as ethanol, methanol, isopropanol, acetonitrile, and acetone at a different percentage with water (vol/vol) on antioxidant efficiency as well as the total phenolic content (TPC) of PDB extracts. Among the fourteen different PDB extracts, the extracts obtained from 20% ethanol (E20), 40% ethanol (E40), and 20% acetonitrile (ACN20) showed more significant antioxidant potential, as well as high total phenol content (TPC). Extracts from other aqueous mixtures of organic solvents such as isopropanol, acetone, and methanol, as well as water, showed lesser antioxidant capacity and also had less TPC compared to these three most active extracts, E20, E40, and ACN20. Moreover, using ethanol at 100% for extraction significantly decreased the TPC and antioxidant capacity of PDB extracts. Data are implicating that an increased phenolic content in PDB extracts proportionally increases their antioxidant efficiency.

## 1. Introduction

Pine is an evergreen, coniferous, resinous, extended living, and mostly monoecious tree. They are very native to the earth northern hemisphere, including Korea [1]. There are reports saying that at least a hundred pine species exist in Korea [2]. The most common Korean native pine species are* Pinus densiflora*,* Pinus thunbergii*, and* Pinus koraiensis*. These native pine species, along with some exotic species, have always been primarily considered in Korea forestry plantations [3]. Thus, at present, pine has occupied approximately 52%, among which 42% by* Pinus densiflora*, of total forest area in Korea [4]. As pine woods are sturdy and durable, they are often utilized for the general household construction process in Korea. Presently, wood industries from Korea are producing approximately 300 tonnes of pine bark as by-product every year and are always treated as waste with little to no value. Local people freely access this bark waste from industries for using in boilers during the winter season.

It is well known that many phenolic compounds can act as an antioxidant. Phenolic compounds can exert their antioxidant activity by directly reacting with the free radicals and thereby producing a less reactive radical species or terminating the free radical chain reaction. They can also hinder the free radical-mediated chain reactions by indirect mechanisms like regenerating primary antioxidants by donating their hydrogen or electron atom, deactivating the singlet oxygen, or sequestrating triplet oxygen, decomposing the hydroperoxides to nonradical species, chelating metal ions that are responsible for the generation of radicals, and absorbing UV radiation [5]. Hence, there is a strong belief that regular consumption of plant products that are rich in phenolic compounds can reduce the risk of development of oxidative stress-mediated disease complications [6]. Therefore, worldwide, attention has been increased towards the finding of phenolic content-rich new and safe plant sources for utilization in the food and pharmaceutical industries. Since pine bark is well known to have various bioactive compounds including polyphenols, recently a Korean pharmaceutical and food industry, Nutrapharm Co., Ltd., has started making cosmetic and food supplements from* Pinus densiflora* bark (PDB) and selling them in the local market. However, the characteristics of most phenolic substances range from polar to nonpolar by nature; many extraction factors, mainly the solvent system, are very challenging for their extraction efficiency [7–9]. Mostly aqueous mixtures of organic solvents are considered as useful solvents for extraction of polyphenolic compounds from plant materials. Our study provides information on the effect of aqueous mixtures of some of the class 2 (methanol and acetonitrile) and class 3 (ethanol, isopropanol, and acetone) residual solvents, which are recommended by the United States Food and Drug Administration (US FDA), and also suggests the most suitable nontoxic solvent for maximum recovery of phenolic substances and thereby antioxidant-rich product from the PDB.

## 2. Materials and Methods

### 2.1. Reagents

The solvents used in this study such as ethanol, methanol, acetone, acetonitrile, and isopropanol were of analytical or HPLC grade. The chemicals, 2,2-diphenyl-1-picrylhydrazyl (DPPH, Cat. No. D9132), 2,2′-azino-bis(3-ethylbenzthiazoline-6-sulfonic acid) (ABTS, Cat. No. 11557), (±)-6-hydroxy-2,5,7,8-tetramethylchromane-2-carboxylic acid (Trolox, Cat. No. 238813), potassium persulfate (Cat. No. 216224), gallic acid (Cat. No. G7384), hydrogen peroxide (Cat. No. H1009), and Folin–Ciocalteu reagent (Cat. No. 47641), were purchased from Sigma Chemical Company, St. Louis, USA. The 2,4,6-Tris(2-pyridyl)-1,3,5-triazine (TPTZ, Cat. No. T0530) and 1,10-phenanthroline (Cat. No. P1826) were obtained from Tokyo Chemical Industry Co., Japan. Ascorbic acid (Cat. No. 01452) was from Kanto Chemical Co., Japan. All other chemicals used in our study were of analytical grade from local companies.

### 2.2. Plant Material Collection and Extraction

*Pinus densiflora* bark (PDB) was obtained from the National Forestry Cooperative Federation, Donghae City, Korea. The identity of the plant was confirmed, and the voucher specimen (PDB/2018) was deposited in the College of Science and Technology, Kookmin University, Korea. The bark was dried in the shade at room temperature for about a week. The dried bark was chopped and subjected to extraction for 9 h at 60°C, using ethanol in the range of 0, 20, 40, 60, 80, or 100%, as well as 20 or 40% methanol, isopropanol, acetonitrile, and acetone with distilled water (vol/vol) as an extraction solvent. The proper ratio of plant material to extraction solvent, extraction time, and temperature was analyzed previously and followed here. A six-port heating mantle (EAM-9202-06, 250 ml capacity, 150 WATTS, Medline Scientific Limited, Oxon, UK, OX44 7XZ) was used for extraction. All the extracts were filtered using Whatman filter paper No. 1 and concentrated under reduced pressure and 50°C using a rotary evaporator. The stock solution of extracts was prepared using dimethyl sulfoxide (DMSO) of 99.9% purity (Sigma, D8418), at a concentration of 20 mg/mL, and used for further experimental analysis.

### 2.3. Determination of Total Phenol Content

The total phenolic content of pine bark extracts was quantified by the Folin-Ciocalteu method as described previously with some modifications [10]. Briefly, 0.8 mL of PDB extract at 50 *μ*g/mL concentration in distilled water was thoroughly mixed with 0.1 mL of 1:4 diluted Folin-Ciocalteu reagent and incubated for 3 min at room temperature. Afterward, 0.1 mL of 10% sodium carbonate was added to each sample mixture and was allowed to stand for a further 30 min in the dark at room temperature. Then, 0.1 mL from each sample was transferred into a 96-well plate, and the absorbance at 760 nm was measured using a multimode plate reader. Gallic acid in the range of 0-20 *μ*g/mL was used as a reference standard. Data were expressed as gallic acid equivalent (GAE) mg/g of PDB extract.

### 2.4. Liquid Chromatography/Mass Spectrometry (LC/MS) Analysis

The concentrated PDB extracts were dissolved in ethanol or methanol to water (1:1) at 100 mg/mL concentration. The undissolved solid contents in the samples were removed by centrifugation at 1000 rpm for 10 min. The supernatant of the samples was passed through a syringe filter (PVDF membrane, 0.2 *μ*m pore size, 13 mm diameter) and the resulting filtrate was used for liquid chromatography/mass spectrometry (LC/MS) analysis. LC was run in 2795 separation module (Agilent, California, USA) with Photodiode Array 996 detector (Waters, Massachusetts, USA) using a Halo 90 Å C18 column, 2.1 × 150 mm, 5 *μ*m (Advanced Materials Technology, USA). The detection wavelength was 210-400 nm. The sample injection volume was 10 *μ*L, and the mobile phases used were of 0.1% trifluoroacetic acid with water (solvent A), and 0.1% trifluoroacetic acid with acetonitrile (solvent B) at different gradient conditions and a flow rate of 0.2 mL/min. The gradient conditions of the mobile phases used for the analysis were depicted in Table 1. MS was performed on Waters Micromass Quattro micro API equipped with ESI scanning mode. MS spectra were recorded in positive ionization mode between m/z 100 and 1000.

### 2.5. DPPH Radical Scavenging Assay

The 10-fold concentrated DPPH radical stock solution (1 mmol/L) was prepared freshly and diluted to 1:10 ratio using aqueous methanol, just before use for the assay. The assay was conducted using a 96-well microplate according to the previous reports with some changes [11]. Briefly, 0.1 mL of PDB extract at 0-100 *μ*g/mL or Trolox at 0-10 *μ*g/mL concentration in aqueous methanol was mixed with 0.1 mL of DPPH radical solution (0.1 mmol/L). The reaction mixtures were incubated in the dark at room temperature for 10 min, and then the decrease in absorbance at 515 nm was measured using a multimode plate reader. Data were expressed as Trolox equivalent (TE) mg/g of PDB extract.

### 2.6. Total Antioxidant Capacity (TAC) Assay

The total antioxidant capacity of PDB extracts was determined by the colorimetric ammonium molybdate method, as described previously with slight changes [12]. In brief, all PDB extracts were diluted to 200 *μ*g/mL concentration using distilled water. Then the diluted extract (0.1 mL) was transferred into a 2.0 mL capacity Eppendorf tubes and to which 0.9 ml of assay mixture made of 0.6 mol/L sulfuric acid, 28 mmol/L sodium phosphate, and 4 mmol/L ammonium molybdate was added and incubated in a water bath at 90°C for 90 min. The samples were left to cool down to room temperature and were centrifuged at 5000 rpm for 5 min. The supernatants (0.1 mL) were carefully transferred into a 96-well plate, and the absorbance of the samples was measured at 695 nm. Ascorbic acid at a concentration of 0-125 *μ*g/mL was used to prepare a standard curve. The activity of extracts was expressed as ascorbic acid equivalent (AAE) mg/g of PDB extract.

### 2.7. ABTS Radical Scavenging Assay

First, the ABTS radical stock solution was prepared by mixing an equal volume of 7 mmol/L ABTS and 2.45 mmol/L of potassium persulfate aqueous solutions and incubating the same for 12-16 h under dark condition. The ABTS stock solution was diluted with aqueous methanol (1:33) and then used for the determination of radical scavenging potential of PDB extracts [13]. Briefly, 0.1 mL of PDB extract at 0-100 *μ*g/mL or Trolox at 0-10 *μ*g/mL concentration in aqueous methanol was added to an equal volume of diluted ABTS radical solution and incubated for 10 min at room temperature in the dark. The decrease in absorbance of the reaction samples at 734 nm was measured, and the activity of extracts was expressed as Trolox equivalent (TE) mg/g of PDB extract.

### 2.8. Ferric-Reducing Antioxidant Power (FRAP) Assay

The assay was done by mixing 50 *μ*L of PDB extracts (100 *μ*g/mL), aqueous methanol for blank or ascorbic acid (1.25-40 *μ*g/mL) as a reference, with 200 *μ*L of FRAP reagent [14]. The mixture was incubated for 5 min at 37°C, and the absorbance at 620 nm was observed. The FRAP reagent was made by mixing the following three different solutions, 10 mmol/L 2,4,6-tri(2-pyridyl)-1,3,5-triazine (TPTZ), 300 mmol/L acetate buffer of pH 3.6, and 20 mmol/L FeCl_3_ at 1:10:1 ratio, just before the assay. Data were expressed as ascorbic acid equivalent (AAE) mg/g of PDB extract.

### 2.9. H_2_O_2_ Scavenging Assay

This assay was performed as per the method described by Debanjan Mukhopadhyay et al. with some changes [15]. The reaction mixture consisting of 100 *μ*L of FeSO_4_ (1.5 mmol/L), 100 *μ*L of PDB extract (1 mg/mL) or gallic acid (6.25 to 100 *μ*g/mL) or aqueous methanol (for blank), and 100 *μ*L of H_2_O_2_ (1 mmol/L) was incubated at room temperature for 5 min, in the dark. Then, 600 *μ*L of 1,10-phenanthroline (1.5 mmol/L) was added to each reaction mixture and kept at room temperature for another 10 min, in the dark. The samples were centrifuged, supernatants were transferred into a 96-well plate, and the absorbance was recorded at 536 nm through a multimode plate reader. Blanks with and without H_2_O_2_ were utilized for the assay. Data were expressed as gallic acid equivalent (GAE) mg/g of PDB extract.

### 2.10. Statistical Analysis

The experimental data were subjected to analysis using statistical GraphPad Prism software. Data are presented as the mean ± SD of triplicate assays. The significant difference between the experimental groups was determined by one-way analysis of variance (ANOVA) followed by Sidak test. The *p*-value less than 0.05, 0.01, and 0.001 was considered statistically significant.

## 3. Results and Discussion

After the extraction was done using different solvents, the resulting PDB extracts were concentrated under reduced pressure, and the percentage yield from each extract was calculated against the initial amount of the solid material. As shown in Table 2, the extraction solvent 40% isopropanol with water (vol/vol) gave a maximum yield compared to all other solvents used in our study.

**Figure 1:**
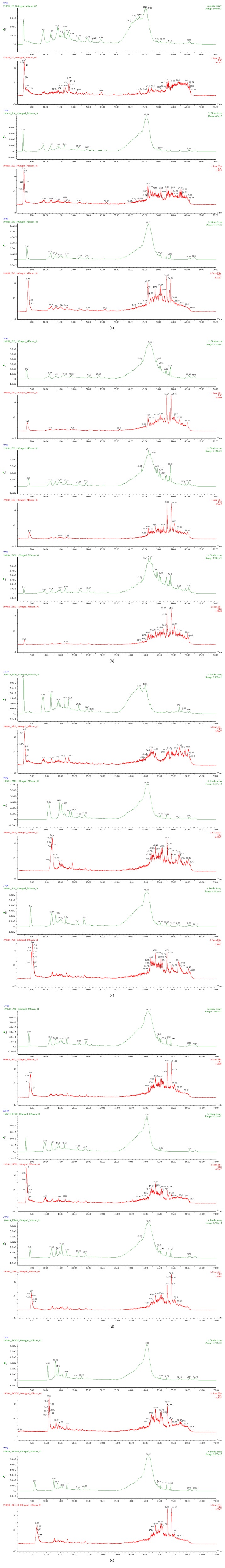
LC/MS profile of* Pinus densiflora* bark (PDB) extracts from different solvents.

A typical LC/MS profile of PDB extracts is presented in Figures 1(a)–1(e). The results show that there are some peak differences between different solvent extracts. However, further detailed analysis is necessary to elucidate the exact compositional difference between the extracts.

### 3.1. Effect of Extraction Solvents on Total Phenol Content of PDB Extracts

Phenolic compounds are secondary metabolites produced by plants in response to various environmental stresses such as ultraviolet radiation and pathogens [16]. These compounds can neutralize the free radicals by their multifunctional properties such as hydrogen donating, reducing, metal chelating, and singlet oxygen quenching properties [17–19]. They also terminate the free radical chain reaction by forming a relatively stable phenoxy-radical intermediate [20]. Hence, the antioxidant property of plant extracts is generally attributed to phenolic compounds. Nowadays, regarding food, there is a tendency to consume safer and healthy products that should have at least some amount of natural antioxidants. Hence, in food industries, attention has been increased in the isolation of phenolic compounds from plant sources to be used as a natural antioxidant supplement in various food products [21]. There are many factors that play a critical role in the successful isolation of phenolic compounds with potent antioxidant activity from plant materials. Among them, the choice of solvent is the most significant [22]. Numerous reports state that aqueous mixtures of organic solvents are the most suitable for extraction of phenolic compounds from plant sources. Different plant material requires different solvent type for maximum extraction of phenolic compounds. Bhebhe et al. (2016) have reported that acetone at 50% with water could extract the highest TPC from* Camellia sinensis*,* Lippia javanica*, and* Ilex paraguariensis*, whereas, 50% of ethanol with water is required to get the highest TPC from* Cuphea carthagenensis*  [22]. Based on this background and many other reports, in the present study, we employed extraction of PDB to get maximum TPC using an aqueous mixture of some of class 2 (methanol and acetonitrile) and class 3 (ethanol, acetone, and isopropanol) residual organic solvents which are recommended by the US FDA. Moreover, previously, we found the extraction of PDB at 60°C for more than 9 h did not significantly increase the TPC of the extracts. Further, the TPC of the extracts was significantly decreased when extraction was performed at temperature 70°C or above, time dependently. Hence, in the present study, we performed the extraction of PDB for 9 h at a minimum temperature of 60°C.  Table 3 shows the TPC of PDB extracts from different solvents. The first and highest TPC level belongs to the extract E20 (266.05 ± 4.30 mg GAE/g PDB extract), followed by ACN20 extract (257.68 ± 6.14 mg GAE/g PDB extract), E40 extract (255.57 ± 4.44 mg GAE/g PDB extract), ISP40 extract (244.49 ± 1.94 mg GAE/g PDB extract), and the other 11 different PDB extracts. The TPC of aqueous water extract E0 was 146.35 ± 0.01 mg GAE/g PDB extract, which is approximately 45% lesser than the extract E20 that has the highest TPC. The PDB extract obtained from 100% ethanol showed the lowest TPC (92.75 ± 2.04 mg GAE/g PDB extract) compared to all other different PDB extracts.

Table 4 represents the correlation between the total phenol content and antioxidant activity of PDB extracts. A correlation analysis was performed using statistical software GraphPad Prism, according to instructions. The results showed that the correlation coefficient was significant (*∗∗∗p < 0.001*) for all the determined antioxidant assays. This substantiates that the antioxidant activity of PDB extracts is due to the presence of phenolic antioxidant compounds.

### 3.2. Effect of Extraction Solvents on Antioxidant Quality of PDB Extract

Many existing studies clearly state that instead of water alone the use of aqueous mixtures of organic solvents like ethanol, methanol, acetone, isopropanol, or acetonitrile with water for extraction greatly increases the antioxidant efficiency of most plant products [23–26]. Based on this background and considering the hazards of solvents, we first determined the effect of ethanol, which is considered much safer next to the water for human consumption, on the antioxidant quality of bark extracts. Ethanol was used at 0, 20, 40, 60, 80, and 100% with water (vol/vol) for extraction. According to our previous study, the ratio of plant material to solvent was fixed at 1:10 proportion (200 mL for 20 g of bark) and extraction was done at 60°C for 9 h. All the ethanolic extracts were first subjected to DPPH radical assay. The results from this assay explored that the activity of PDB extracts (data not shown) declined in the order: E40>E20>E60>E80>E0>E100. The extracts obtained from ethanol at 20 or 40% with water (vol/vol) for extraction gave the highest free radical scavenging potential. However, extract from absolute ethanol (100%) showed the lowest activity compared to all other ethanolic PDB extracts as well as PDB water extract. From this, we assumed that aqueous mixtures of organic solvents more than 40% with water (vol/vol) might not be efficient for the extraction of antioxidant-rich PDB extract. Thus we limited the maximum percentage of all other extraction solvents methanol, acetone, isopropanol, and acetonitrile to 40% with water (vol/vol) to determine their influence on the antioxidant quality of PDB extract and for comparing with the ethanolic PDB extracts.

**Figure 2:**
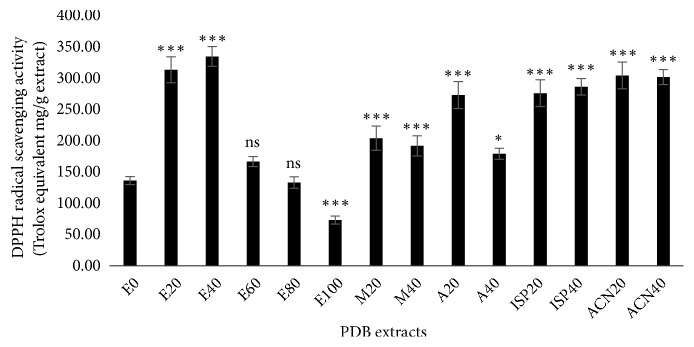
DPPH radical scavenging activity of* Pinus densiflora* bark (PDB) extracts from different solvents. Activity is expressed as Trolox equivalent (TE) mg/g extract. Values are mean ± SD from three independent experiments. *∗p < 0.05*, *∗∗∗p < 0.001*,* ns: nonsignificant*, compared to E0 (water extract).

Several* in vitro* chemical assays with different reaction principles have been frequently used to determine the antioxidant potential of plant extracts. DPPH radical assay is simple and most common by which we can quickly understand the antioxidant efficiency of any compound, including plant extracts in a short time. DPPH is a purple color stable organic radical which absorbs light at 515 nm. After reduction by accepting the hydrogen atom from antioxidants the purple color of DPPH changes to yellow and therefore loses its absorbance capacity at 515 nm. Hence, by measuring a decrease in absorbance at 515 nm as a result of the reduction of DPPH radical, it is possible to determine the antioxidant potential of plant extracts [27].  Figure 2 shows the DPPH radical scavenging potential of different PDB extracts in Trolox equivalent (TE) mg/g of extract. The extracts obtained from ethanol (E20 and E40) showed the highest radical scavenging activity. The solvents acetonitrile and isopropanol at 20 and 40% also strongly improved the antioxidant potential of PDB extracts next to ethanolic extracts (E20 and E40). Further, it was evident that ethanol at 100% markedly reduced the antioxidant capacity of PDB extracts.

**Figure 3:**
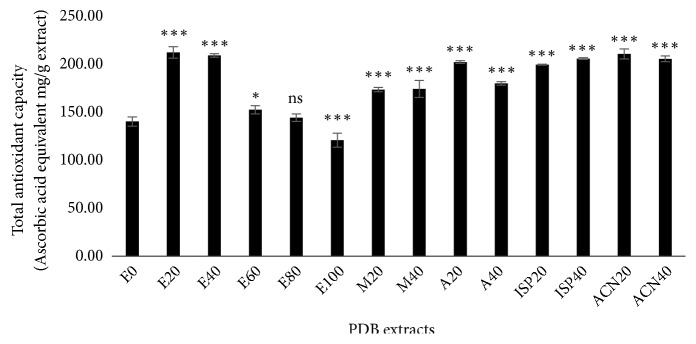
Total antioxidant capacity (TAC) of* Pinus densiflora* bark (PDB) extracts from different solvents. Activity is expressed as ascorbic acid equivalent (AAE) mg/g extract. Values are mean ± SD from three independent experiments. *∗p < 0.05*, *∗∗∗p < 0.001*,* ns: nonsignificant, *compared to E0 (water extract).

It is evident that the same antioxidant molecule may act differently in the scavenging of different types of radicals. Hence, we performed additional scavenging assays such as TAC, ABTS, FRAP, and H_2_O_2_ to explore the antioxidant efficiency of PDB extracts further. The total antioxidant capacity (TAC) of extracts was determined based on the principle of reduction of molybdenum (VI) to molybdenum (V) by antioxidants and then the formation of phosphomolybdenum (V) complex at acidic reaction condition. The resulting complex is green in color and absorbs light at 695 nm [28]. Ascorbic acid was used as a reference antioxidant compound.  Figure 3 shows the TAC of different PDB extracts. The results were expressed as ascorbic acid equivalent (AAE) mg/g of PDB extract. Among the fourteen different extracts, the 20% ethanol, 20% acetonitrile, and 40% ethanol extracts of bark showed the maximum TAC. The 100% ethanolic extract showed the lowest TAC compared to all other extracts. The most active 20% and 40% ethanolic extracts and 20% acetonitrile extract showed approximately 1.5-fold increased TAC compared to water extract. Though the methanol, acetone, and isopropanol extracts showed better TAC compared to water extract, still it was lesser than the activity of both 20 and 40% ethanolic PDB extracts.

**Figure 4:**
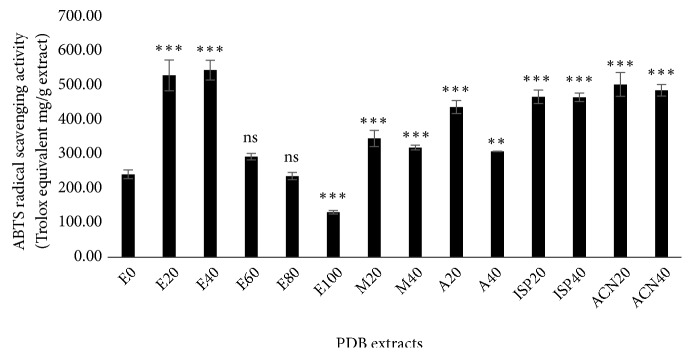
ABTS radical scavenging activity of* Pinus densiflora* bark (PDB) extracts from different solvents. Activity is expressed as Trolox equivalent (TE) mg/g extract. Values are mean ± SD from three independent experiments. *∗∗p < 0.01*, *∗∗∗p < 0.001*,* ns: nonsignificant*, compared to E0 (water extract).

Like DPPH, ABTS is also a chemical radical not found in the human system. When a solution of ABTS radical is mixed with an antioxidant that can donate a hydrogen atom, the blue-green color of the radical solution becomes colorless and loses absorbance at 734 nm. Hence, it is considered that the percentage loss of color intensity is equal to the radical scavenging ability of antioxidants [29]. Trolox was used as a reference antioxidant, and the activity of PDB extracts was expressed as Trolox equivalent (TE) mg/g extract. The degree of reduction of ABTS radical by different PDB extracts (Figure 4) declined in the order: E40, E20, ACN20, ACN40, ISP20, ISP40, A20, M20, M40, A40, E60, E0, E80, and lastly E100. Here we could understand that extraction of PDB using aqueous mixtures of ethanol either at 20 or at 40% with water markedly increases the antioxidant potential compared to the extracts from the same solvent of ethanol at 60-100%, other solvents acetonitrile, isopropanol, acetone, and methanol at 20 or 40% with water, as well as aqueous water.

**Figure 5:**
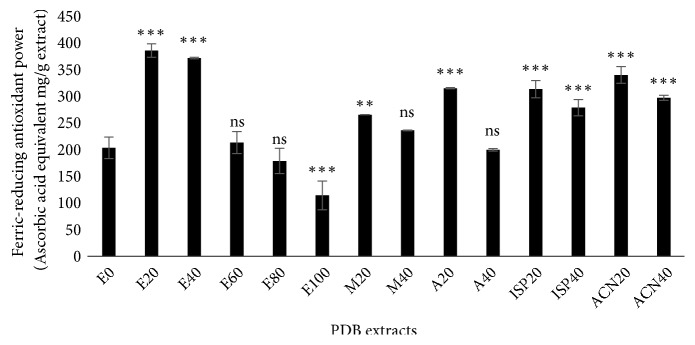
Ferric-reducing antioxidant power (FRAP) of* Pinus densiflora* bark (PDB) extracts from different solvents. Activity is expressed as ascorbic acid equivalent (AAE) mg/g extract. Values are mean ± SD from three independent experiments. *∗∗p < 0.01*, *∗∗∗p < 0.001*,* ns: nonsignificant*, compared to E0 (water extract).

FRAP assay is based on the principle of reduction of a ferric form of the Fe^3+^-TPTZ complex to the ferrous form Fe^2+^-TPTZ by antioxidants in acidic condition, where the yellow color of the reaction mixture turns to green-blue with various intensities depending on the reducing power of antioxidants [30]. Hence, by measuring the increase in absorbance at 593 nm, we can explore the reducing strength of antioxidants. In our study (Figure 5), we found the PDB extracts E20, E40, and ACN20 gave the highest reducing power than other PDB extracts, including PDB water extract. Once again, the lowest activity was found in the PDB extract obtained from 100% ethanol.

**Figure 6:**
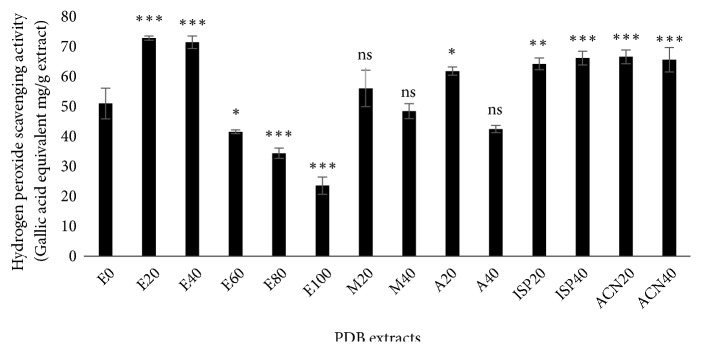
Hydrogen peroxide (H_2_O_2_) scavenging potential of* Pinus densiflora* bark (PDB) extracts from different solvents. Activity is expressed as gallic acid equivalent (GAE) mg/g extract. Values are mean ± SD from three independent experiments. *∗p < 0.05*, *∗∗p < 0.01*, *∗∗∗p < 0.001*,* ns: nonsignificant*, compared to E0 (water extract).

In the human body, cellular mitochondria continuously produce superoxide anion radical and subsequently hydrogen peroxide (H_2_O_2_) at a certain level through electron transport chain [31, 32]. Additionally, the human body is daily exposed to H_2_O_2_ from various environmental sources, mostly from leaf crops. There are reports indicating that approximately 0.28 mg of H_2_O_2_/kg/day enters into the human body through inhalation, eye, or skin contact [33]. H_2_O_2_ can cross the cell membrane easily, but it is poorly reactive and cannot oxidize biological molecules. However, the danger of H_2_O_2_ comes from its decomposition product the highly reactive hydroxyl radical as a result of exposure to UV or interaction with the transition metal ions mostly iron in the human system [34]. So it is apparent that increased levels of H_2_O_2_ indirectly damage tissues. In the present study, we determined the efficiency of different PDB extracts in the scavenging of H_2_O_2_ molecule. The assay was based on the principle of formation of a red-orange complex between ferrous iron (Fe^2+^) and 1,10-phenanthroline that absorb light at 536 nm. While antioxidants fail to scavenge H_2_O_2_, H_2_O_2_ can oxidize ferrous (Fe^2+^) to ferric (Fe^3+^) iron which is incapable of forming a complex. Hence, the amount of red-orange compound produced in the assay mixture is directly proportional to the H_2_O_2_ scavenging potential of antioxidants [15]. We found (Figure 6) that both E20 and E40 PDB extracts significantly increased the formation of red-orange ferrous iron (Fe^2+^) and 1,10-phenanthroline complex evidenced from increased absorbance at 536 nm compared to extracts from other solvents as well as E0, E60, E80, and E100. Data depicts that E20 and E40 extracts have more significant H_2_O_2_ scavenging potential. Conversely, E100 showed the lowest activity compared to all other PDB extracts.

## 4. Conclusion

The results of the antioxidant assays DPPH and ABTS indicated that the PDB extracts obtained from 40% ethanol have the highest antioxidant activity, whereas TAC, FRAP, and H_2_O_2_ scavenging assays showed that the 20% ethanolic extract is better than 40% ethanolic extract. Further, the PDB extract from 20% acetonitrile showed very close activity to the 20 and 40% ethanolic extracts in all the antioxidant assays performed in our study. The PDB extracts, from water, and aqueous mixtures of isopropanol, acetone, and methanol showed less antioxidant activity compared to the most active PDB extracts E20, E40, and ACN20. We found the extraction of PDB using ethanol at 100% significantly decreased the antioxidant activity compared to water extract. Moreover, we observed that increased antioxidant potential of PDB extracts directly correlates with an increased amount of TPC. By considering the antioxidant activity and total phenol content of extracts, and solvent toxicity to human and environment, our study suggests that ethanol at 20 or 40% with water (vol/vol) can be used for extraction of PDB to improve the yield of total phenol content and thereby antioxidant capacity.

## Figures and Tables

**Table 1:** 

Time (min)	A (%)	B (%)
0.00	100.0	0.0
3.00	100.0	0.0
10.00	90.0	10.0
30.00	90.0	10.0
40.00	70.0	30.0
50.00	0.0	100.0
55.00	0.0	100.0
60.00	100.0	0.0
70.00	100.0	0.0

**Table 2:** Effect of extraction solvent on the yield of *Pinus densiflora* bark (PDB) extract.

Plant Material	Extraction Solvent	Extract Name	Extract Yield (% solid)
PDB	Water	E0	6.18
PDB	20% ethanol with water (vol/vol)	E20	8.34
PDB	40% ethanol with water (vol/vol)	E40	9.52
PDB	60% ethanol with water (vol/vol)	E60	11.76
PDB	80% ethanol with water (vol/vol)	E80	10.21
PDB	100% ethanol with water (vol/vol)	E100	8.15
PDB	20% methanol with water (vol/vol)	M20	8.36
PDB	40% methanol with water (vol/vol)	M40	9.09
PDB	20% acetone with water (vol/vol)	A20	8.41
PDB	40% acetone with water (vol/vol)	A40	9.58
PDB	20% isopropanol with water (vol/vol)	ISP20	10.11
PDB	40% isopropanol with water (vol/vol)	ISP40	12.05
PDB	20% acetonitrile with water (vol/vol)	ACN20	9.24
PDB	40% acetonitrile with water (vol/vol)	ACN40	11.37

**Table 3:** Effect of extraction solvents on the total phenol content (TPC) of *Pinus densiflora* bark (PDB) extract.

Plant Material	Extraction solvent	Extract Name	TPC (GAE mg/g extract)
PDB	Water	E0	146.35 ± 0.01
PDB	20% ethanol with water (vol/vol)	E20	266.05 ± 4.30^*∗∗∗*^
PDB	40% ethanol with water (vol/vol)	E40	255.57 ± 4.44^*∗∗∗*^
PDB	60% ethanol with water (vol/vol)	E60	171.38 ± 1.09^*∗∗∗*^
PDB	80% ethanol with water (vol/vol)	E80	144.60 ± 3.22^ns^
PDB	100% ethanol with water (vol/vol)	E100	92.75 ± 2.04^*∗∗∗*^
PDB	20% methanol with water (vol/vol)	M20	200.44 ± 3.52^*∗∗∗*^
PDB	40% methanol with water (vol/vol)	M40	191.14 ± 0.24^*∗∗∗*^
PDB	20% acetone with water (vol/vol)	A20	240.89 ± 0.11^*∗∗∗*^
PDB	40% acetone with water (vol/vol)	A40	179.13 ± 1.51^*∗∗∗*^
PDB	20% isopropanol with water (vol/vol)	ISP20	242.47 ± 0.92^*∗∗∗*^
PDB	40% isopropanol with water (vol/vol)	ISP40	244.49 ± 1.94^*∗∗∗*^
PDB	20% acetonitrile with water (vol/vol)	ACN20	257.68 ± 6.14^*∗∗∗*^
PDB	40% acetonitrile with water (vol/vol)	ACN40	243.48 ± 0.51^*∗∗∗*^

Values are expressed as Gallic acid equivalent (GAE) mg/g extract. Values are mean ± SD from three independent experiments. *∗∗∗p < 0.001*, *ns-nonsignificant*, compared to E0 (water extract).

**Table 4:** Correlation between total phenol content (TPC) and antioxidant activity of *Pinus densiflora* bark (PDB) extracts.

Correlation analysis	TPC vs. DPPH	TPC vs. TAC	TPC vs. ABTS	TPC vs. FRAP	TPC vs. H_2_O_2_
Pearson r					
r	0.9857	0.9834	0.9877	0.9507	0.9497
R squared	0.9715	0.9672	0.9756	0.9039	0.9019
P value	<0.001	<0.001	<0.001	<0.001	<0.001
P value summary	*∗∗∗*	*∗∗∗*	*∗∗∗*	*∗∗∗*	*∗∗∗*
Significant? (alpha = 0.05)	Yes	Yes	Yes	Yes	Yes

## Data Availability

The data used to support the findings of this study are included in the manuscript.

## Supplementary Materials

Supplementary Materials Supplementary Table 1: the IC_50_ (*μ*g/mL) value of* Pinus densiflora* bark (PDB) extracts for three different antioxidant assays.

## References

[1] Global Invasive Species Database, Species profile: Pinus, 2019

[2] Korea Forest Research Institute, Economic tree species: Pine tree, 2012

[3] Kim K. H., Zsuffa L. (1994). Reforestation of South Korea: The history and analysis of a unique case in forest tree improvement and forestry. *The Forestry Chronicle*.

[4] Korea Forest Service, Statistical Yearbook of Forestry, 2018

[5] Galano A., Mazzone G., Alvarez-Diduk R., Marino T., Alvarez-Idaboy J. R., Russo N. (2016). Food antioxidants: chemical insights at the molecular level. *Annual Review of Food Science and Technology*.

[6] Pourreza N. (2013). Phenolic compounds as potential antioxidant. *Jundishapur Journal of Natural Pharmaceutical Products*.

[7] Do Q. D., Angkawijaya A. E., Tran-Nguyen P. L. (2014). Effect of extraction solvent on total phenol content, total flavonoid content, and antioxidant activity of Limnophila aromatica. *Journal of Food and Drug Analysis*.

[8] Uzel R. A. (2018). Effect of extraction method and extraction solvent on recovery of phenolic compounds from olive leaves in Kemalpaşa-İzmir (Turkey): Oleuropein recovery as a case example. *Separation Science and Technology*.

[9] Alcântara M. A., de Lima Brito Polari I., de Albuquerque Meireles B. R. (2019). Effect of the solvent composition on the profile of phenolic compounds extracted from chia seeds. *Food Chemistry*.

[10] Okmen B., Sigva H. O., Mutlu S., Doganlar S., Yemenicioglu A., Frary A. (2009). Total antioxidant activity and total phenolic contents in different turkish eggplant (Solanum Melongena L.) cultivars. *International Journal of Food Properties*.

[11] Liu C. J., Zhang S. Q., Zhang J. S., Liang Q., Li D. S. (2012). Chemical composition and antioxidant activity of essential oil from berries of Schisandra chinensis (Turcz.) Baill. *Natural Product Research*.

[12] Wan C., Yu Y., Zhou S., Liu W., Tian S., Cao S. (2011). Antioxidant activity and free radical-scavenging capacity of Gynura divaricata leaf extracts at different temperatures. *Pharmacognosy Magazine*.

[13] Sowndhararajan K., Kang S. C. (2013). Free radical scavenging activity from different extracts of leaves of Bauhinia vahlii Wight & Arn. *Saudi Journal of Biological Sciences*.

[14] Pulido R., Bravo L., Saura-Calixto F. (2000). Antioxidant activity of dietary polyphenols as determined by a modified ferric reducing/antioxidant power assay. *Journal of Agricultural and Food Chemistry*.

[15] Mukhopadhyay D., Dasgupta P., Sinha Roy D. (2016). A sensitive in vitro spectrophotometric hydrogen peroxide scavenging assay using 1,10-phenanthroline. *Free Radicals and Antioxidants*.

[16] Yang L., Wen K. S., Ruan X., Zhao Y. X., Wei F., Wang Q. (2018). Response of plant secondary metabolites to environmental factors. *Molecules*.

[17] Chun O. K., Kim D., Lee C. Y. (2003). Superoxide radical scavenging activity of the major polyphenols in fresh plums. *Journal of Agricultural and Food Chemistry*.

[18] Choe E., Min D. B. (2009). Mechanisms of antioxidants in the oxidation of foods. *Comprehensive Reviews in Food Science and Food Safety*.

[19] Mathew S., Abraham T. E., Zakaria Z. A. (2015). Reactivity of phenolic compounds towards free radicals under in vitro conditions. *Journal of Food Science and Technology*.

[20] Leopoldini M., Marino T., Russo N., Toscano M. (2004). Antioxidant properties of phenolic compounds: H-atom versus electron transfer mechanism. *The Journal of Physical Chemistry A*.

[21] Caleja C., Ribeiro A., Barreiro M. F., Ferreira I. C. (2017). Phenolic compounds as nutraceuticals or functional food ingredients. *Current Pharmaceutical Design*.

[22] Bhebhe M., Füller T. N., Chipurura B., Muchuweti M. (2016). Effect of solvent type on total phenolic content and free radical scavenging activity of black tea and herbal infusions. *Food Analytical Methods*.

[23] Zhao H., Dong J., Lu J. (2006). Effects of extraction solvent mixtures on antioxidant activity evaluation and their extraction capacity and selectivity for free phenolic compounds in barley (Hordeum vulgare L.). *Journal of Agricultural and Food Chemistry*.

[24] Jun H., Song G., Yang E., Youn Y., Kim Y. (2012). Antioxidant activities and phenolic compounds of pigmented rice bran extracts. *Journal of Food Science*.

[25] Dailey A., Vuong Q. V. (2015). Effect of extraction solvents on recovery of bioactive compounds and antioxidant properties from macadamia (Macadamia tetraphylla) skin waste. *Cogent Food & Agriculture*.

[26] Mokrani A., Madani K. (2016). Effect of solvent, time and temperature on the extraction of phenolic compounds and antioxidant capacity of peach (Prunus persica L.) fruit. *Separation and Purification Technology*.

[27] Kedare S. B., Singh R. P. (2011). Genesis and development of DPPH method of antioxidant assay. *Journal of Food Science and Technology*.

[28] Prieto P., Pineda M., Aguilar M. (1999). Spectrophotometric quantitation of antioxidant capacity through the formation of a phosphomolybdenum complex: specific application to the determination of vitamin E. *Analytical Biochemistry*.

[29] Re R., Pellegrini N., Proteggente A., Pannala A., Yang M., Rice-Evans C. (1999). Antioxidant activity applying an improved ABTS radical cation decolorization assay. *Free Radical Biology & Medicine*.

[30] Benzie I. F., Strain J. J. (1996). The ferric reducing ability of plasma (FRAP) as a measure of ‘antioxidant power’: the FRAP assay. *Analytical Biochemistry*.

[31] Nulton-Persson A. C., Szweda L. I. (2001). Modulation of mitochondrial function by hydrogen peroxide. *The Journal of Biological Chemistry*.

[32] Bao L., Avshalumov M. V., Patel J. C. (2009). Mitochondria are the source of hydrogen peroxide for dynamic brain-cell signaling. *The Journal of Neuroscience*.

[33] Alam M. N., Bristi N. J., Rafiquzzaman M. (2013). Review on in vivo and in vitro methods evaluation of antioxidant activity. *Saudi Pharmaceutical Journal*.

[34] Halliwell B., Clement M. V., Long L. H. (2000). Hydrogen peroxide in the human body. *FEBS Letters*.

