# Supplementary material for: Impact of Different Extraction Solvents on Phenolic Content and Antioxidant Potential of Pinus densiflora Bark Extract
Source: Biomed Res Int. 2019 Jul 29;2019:3520675. doi: 10.1155/2019/3520675 (PMC6699328; doi:10.1155/2019/3520675)
Supplement: Supplementary Materials — Supplementary Table 1: the IC50 (μg/mL) value of Pinus densiflora bark (PDB) extracts for three different antioxidant assays. [file 3520675.f1.docx]

**Suppl. Table 1.** The IC50 (µg/mL) value of *Pinus densiflora* bark (PDB) extract for three different antioxidant assays.

| Extracts | IC50 (DPPH) | IC50 (ABTS) | IC50 (H2O2) |
| --- | --- | --- | --- |
| E0 | 39.14 ± 0.42 | 18.22 ± 2.25 | 1791.50 ± 207.18 |
| E20 | 17.05 ± 0.66 | 8.35 ± 1.41 | 831.05 ± 115.47 |
| E40 | 15.94 ± 0.18 | 8.07 ± 1.00 | 859.35 ± 147.01 |
| E60 | 32.01 ± 0.37 | 14.98 ± 1.45 | 2291.00 ± 258.80 |
| E80 | 40.17 ± 1.65 | 18.59 ± 2.10 | 3381.00 ± 234.76 |
| E100 | 73.20 ± 4.94 | 33.47 ± 3.43 | 8174.50 ± 333.05 |
| M20 | 26.28 ± 2.06 | 12.75 ± 1.86 | 1462.65 ± 249.40 |
| M40 | 27.91 ± 1.80 | 13.73 ± 1.11 | 1849.50 ± 204.35 |
| A20 | 19.59 ± 1.11 | 10.04 ± 1.12 | 1064.45 ± 128.06 |
| A40 | 29.82 ± 0.42 | 14.21 ± 0.66 | 2387.50 ± 185.97 |
| ISP20 | 19.39 ± 1.07 | 9.40 ± 1.03 | 999.65 ± 99.49 |
| ISP40 | 18.65 ± 0.18 | 9.42 ± 0.82 | 958.30 ± 184.84 |
| ACN20 | 17.57 ± 0.78 | 8.76 ± 1.29 | 953.85 ± 85.06 |
| ACN40 | 17.68 ± 0.01 | 9.02 ± 0.89 | 989.40 ± 237.02 |
